# Assessing the diagnostic accuracy of CT perfusion: a systematic review

**DOI:** 10.3389/fneur.2023.1255526

**Published:** 2023-10-11

**Authors:** Tharani Thirugnanachandran, Sean G. Aitchison, Andy Lim, Catherine Ding, Henry Ma, Thanh Phan

**Affiliations:** Stroke and Ageing Research (STAR), Department of Medicine, School of Clinical Sciences at Monash Health, Monash University, Clayton, VIC, Australia

**Keywords:** computed tomography perfusion, penumbra, infarct, thresholds, parameters

## Abstract

**Background and purpose:**

Computed tomography perfusion (CTP) has successfully extended the time window for reperfusion therapies in ischemic stroke. However, the published perfusion parameters and thresholds vary between studies. Using Preferred Reporting Items for Systematic Reviews and Meta-Analyses of Diagnostic Test Accuracy Studies (PRISMA-DTA) guidelines, we conducted a systematic review to investigate the accuracy of parameters and thresholds for identifying core and penumbra in adult stroke patients.

**Methods:**

We searched Medline, Embase, the Cochrane Library, and reference lists of manuscripts up to April 2022 using the following terms “computed tomography perfusion,” “stroke,” “infarct,” and “penumbra.” Studies were included if they reported perfusion thresholds and undertook co-registration of CTP to reference standards. The quality of studies was assessed using the Quality Assessment of Diagnostic Accuracy Studies-2 (QUADAS-2) tool and Standards for Reporting of Diagnostic Accuracy (STARD) guidelines.

**Results:**

A total of 24 studies were included. A meta-analysis could not be performed due to insufficient data and significant heterogeneity in the study design. When reported, the mean age was 70.2 years (SD+/−3.69), and the median NIHSS on admission was 15 (IQR 13–17). The perfusion parameter identified for the core was relative cerebral blood flow (rCBF), with a median threshold of <30% (IQR 30, 40%). However, later studies reported lower thresholds in the early time window with rapid reperfusion (median 25%, IQR 20, 30%). A total of 15 studies defined a single threshold for all brain regions irrespective of collaterals and the gray and white matter.

**Conclusion:**

A single threshold and parameter may not always accurately differentiate penumbra from core and oligemia. Further refinement of parameters is needed in the current era of reperfusion therapy.

## Introduction

Perfusion imaging has revolutionized hyperacute stroke care, enabling a move beyond the traditional 4.5-h threshold and toward a tissue-based approach to treatment and precision medicine in stroke care (1). Perfusion imaging aims to aid decision-making by identifying the tissue at risk of infarction that is still viable, ‘ischemic penumbra,’ (2) and differentiate it from the tissue that has already infarcted, ‘core.’ Therefore, permitting treatment of those who would benefit from reperfusion therapies and avoiding unnecessary harm. At present, the optimal perfusion parameter to identify ‘core’ is thought to be relative cerebral blood flow (rCBF) < 30%, and a mismatch between this and a time to maximum (Tmax) > 6 s is operationally defined as ischemic penumbra (3). Although these perfusion parameters and thresholds have been used to successfully treat patients in randomized controlled trials in the extended time window (1, 4–6), recent reports have questioned the diagnostic accuracy of rCBF <30% in identifying the volume of infarct (7).

Previous systematic reviews investigating parameters and thresholds have included studies using magnetic resonance perfusion-weighted imaging (MR-PWI) (8, 9) or positron emission tomography (PET) (9) as well as CT perfusion (CTP). A recent meta-analysis (10) examining volumetric and spatial accuracy of CTP for identifying core did not account for the multiple thresholds reported for each parameter (11) and included several studies that failed to perform co-registration of CTP to the reference standard. Co-registration is essential to ensure the accuracy of volumetric analysis as images are aligned in the same coordinate space (12). The purpose of this systematic review is to critically evaluate the diagnostic accuracy of CTP parameters and thresholds to differentiate penumbra from core and oligemia in adult stroke patients.

## Methods

### Search strategy

This systematic review of the literature was performed according to the Preferred Reporting Items for Systematic Reviews and Meta-Analyses of Diagnostic Test Accuracy Studies (PRISMA-DTA) guidelines (13) and the Cochrane Database of Systematic Reviews (14). We conducted a standardized search in Medline, Embase, and the Cochrane Library using the terms “computed tomography perfusion,” “stroke,” “infarct core,” and “penumbra” for relevant studies published in peer-reviewed journals up to April 2022. We screened all titles and abstracts and removed duplication. For each eligible study, full-text papers were obtained. Reference lists from previous systematic reviews and meta-analyses were searched for additional studies. Relevant studies were assessed independently by two authors (T.T., S.A.), and discrepancies were resolved by a third reviewer (T.P.).

### Selection criteria

Original articles were included of studies investigating CTP within 24 h of symptom onset, using different perfusion parameters to define infarcted ‘core’ from the salvageable tissue or ‘penumbra’ and tissue which is hypoperfused but not at risk of infarction, ‘benign oligemia.’ We only included studies that reported results of (1) adult acute ischemic stroke patients; (2) published in English; (3) with sample sizes above five; (4) investigated multiple thresholds; (5) used CTP as the index test; (6) used follow-up MR diffusion-weighted imaging (DWI), MRI-flair, and/or non-contrast CT (NCT) as the reference standard; and (7) co-registered index and reference images. The exclusion criteria included (1) studies using animal models or children and (2) studies using other modalities to assess perfusion such as MR-PWI, single photon emission computed tomography (SPECT), or PET.

### Data extraction and analysis

Using a structured template, the following data were extracted: first author’s name, publication year, study setting and design, number of subjects, baseline demographic data of study population, time between symptom onset and CTP imaging, presence of large vessel occlusion, reference imaging standard, time to reference imaging, use of reperfusion therapies between index and reference test, and time to reperfusion. Information relating to imaging protocol including CTP acquisition, post-processing software, perfusion parameters and thresholds investigated, methods for imaging, and statistical analysis was also collected. Data were extracted independently by two reviewers (T.T. and S.A.), with disagreements resolved by a third reviewer (T.P.). We contacted the corresponding author to obtain further information if data were not easily extractable. Parameters and thresholds with the highest AUC were identified for penumbra and core in each study. If not provided, the Youden index was calculated as the sum of sensitivity and specificity minus one (values range from 0 to 1). In this way, the Youden index summarizes the performance of a test at the optimal cutoff threshold by taking into account the sensitivity and specificity. A Youden index value of less than 0.5 does not meet the optimal performance standard for a diagnostic test (15, 16). For studies that used both acute and delayed MRI to define infarct core, we have only reported results for acute DWI imaging.

### Quality assessment

The Quality Assessment of Diagnostic Accuracy Studies-2 (QUADAS-2) tool (17) was used to assess the methodological quality of included studies. Two different authors assessed the methodological quality of the studies (A.L. and C.D.).

## Results

**Figure 1:**
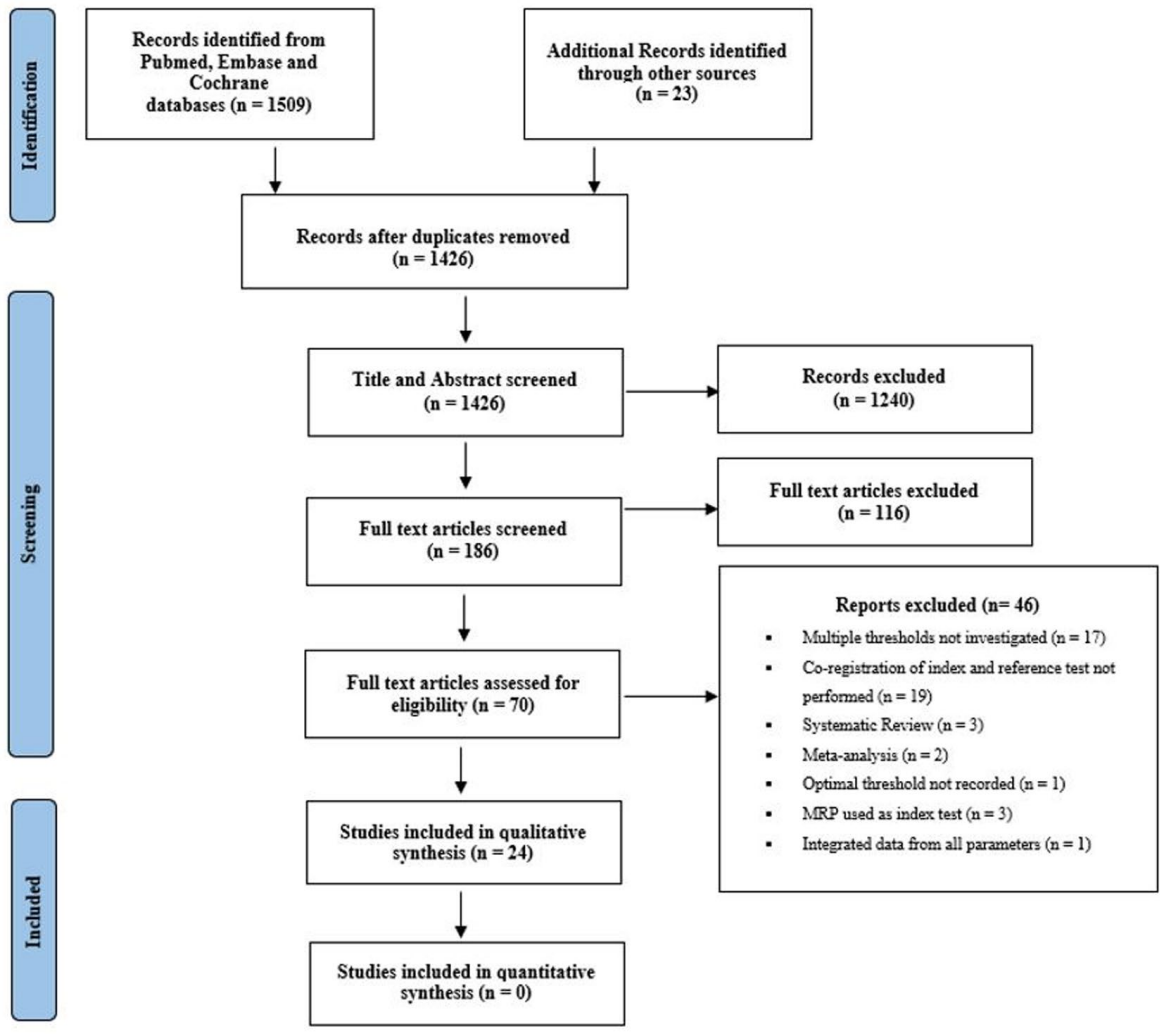
Preferred Reporting Items for Systematic Reviews and Meta-Analyses (PRISMA) flow diagram.

Our MEDLINE, EMBASE, and Cochrane searches yielded 1,509 results. Manual searching yielded an additional 23 studies. We screened 1,426 records using the title and abstract. In total, 186 articles were screened by full text for eligibility. A total of 70 articles were reviewed, and 24 fulfilled the inclusion criteria (see Figure 1). Despite contacting authors, we were unable to determine the exact details of overlapping patients between studies published by the same research groups (18–23). Studies that may have reported results of the same patients were counted once unless they examined different parameters and thresholds for the gray and white matter (24, 25), post-processing software (16, 20, 26–28), brain coverage (19, 23), and the impact of reperfusion therapies (18, 20). A list of excluded studies and reasons for exclusion are available in Supplementary material. Nineteen studies, including three studies that used RAPID® software (29–31), were excluded because the co-registration of index and reference images was not conducted (32–38). Two additional studies using RAPID® software were excluded because they used a combination of MR-PWI and CTP as the index test (39, 40). A meta-analysis was not performed due to insufficient data and significant heterogeneity in methodological characteristics between studies. Results will be discussed under the headings of study design, perfusion parameters, and thresholds.

### Study characteristics

The 24 included studies are shown in Table 1. All were observational cohort studies published between 2006 and 2020. Nine were prospective (21, 22, 24, 25, 41–45). When reported, the mean age was 70.2 years (+/−3.69), and the median NIHSS score on admission was 15, interquartile range (IQR) 13–17. Twenty-three studies investigated core (15, 16, 18–27, 41–51), and 13 studies investigated penumbra (15, 16, 19, 20, 22–25, 28, 41, 43, 49, 50). Most studies included less than 50 patients per study (see Tables 1, 2 in Supplementary material). Of these, it is possible that some studies have included patients from the same period (18–23). When reported, median onset to CTP was 182 (IQR 162–196) min for studies investigating core and 164 (IQR 148–184) min for penumbra (see Tables 1, 2 in Supplementary material). Only 10 studies (51) included patients between 6 and 12 h from stroke onset, and no study reported onset to perfusion imaging beyond 12 h.

**Table 1:** Characteristics of included diagnostic accuracy studies.

Authors	Year	Setting	Start period	End period	Design	Country	Blinding	Scanner	Median onset time to ctp (min)	Scan duration (s)	Contrast injection (mL)	Radiation dose (kvp)	Frame rate (s)	Brain coverage (mm)	Post-processing software	Deconvolution algorithm
Schaefer	2006	Single	2001	2003	Retro	United States	NR	LightSpeed	144	60	45	80	1	20	Advantage workstation	Not specified
Murphy	2006	Multicenter	NR	NR	Pro	Canada	NR	NR	178	45	50	80	1	20	CT Perfusion 3	Deconvolution
Wintermark	2006	Multicenter	NR	NR	Pro	International	NR	NR	240	40–50	40	80–90	1	NR	Philips Medical System	Closed-form non-iterative deconvolution
Murphy	2008	Multicenter	2002	2005	Pro	Canada	Yes	LightSpeed	178	45	50	80	1	20	CT Perfusion 3	Deconvolution
Bivard^a^	2011	Single	2005	2007	Pro	Australia	NR	Phillips	195	45	NR	NR	NR	48	MIStar	Ddsvd
Bivard^b^	2011	Single	2005	2010	Pro	Australia	NR	Phillips	162	60	40	NR	1.33	48–80	MIStar	Ddsvd
Campbell	2011	Single	2003	2007	Pro	Australia	Yes	Phillips	190	45	40	NR	1.3	48	Authors own	Delay-sensitive ssvd
Kamalian	2011	Single	2006	2008	Retro	United States	NR	LightSpeed	246	66	40	80	1	80	CTP “DC” and CTP “Std”	Delay-sensitive svd
Payabvash	2011	Single	2008	2009	Retro	United States	NR	LightSpeed	222^**+**^	90	35	80	3	80	CT Perfusion 3	Deconvolution
Campbell	2012	Single	2003	2007	Pro	Australia	Yes	Phillips	213^**+**^	45	40	NR	1.3	48	Authors own	Delay-sensitive ssvd
Kamalian	2012	Single	2006	2008	Retro	United States	NR	LightSpeed	252	66	40	80	1	80	CTP “DC” and CTP “Std”	Standard and delay corrected svd
Bivard	2013	Single	2005	2011	Retro	Australia	NR	Phillips	162	60	40	NR	1.33	48–80	MIStar	Ddsvd
Bivard	2014	Single	2010	2012	Retro	Australia	NR	Aquilion One	148	65	40	80	NR	160	MIStar	Ddsvd
Eilaghi	2014	Single	NR	NR	Pro	Canada	No	VCT	NR	NR	50	80	1	40	CT Perfusion 4	Delay corrected svd
McVerry	2014	Single	2008	2010	Retro	Scotland	Yes	Phillips Brilliance	180	NR	50	80	2	40	MIStar	Ddsvd
d’Esterre	2015	Multicenter	NR	NR	Pro	International	NR	LightSpeed	193.5	60–90	45	NR	2.8	40–80	CT Perfusion 4D	Delay-insensitive deconvolution
Cereda	2016	Multicenter	2004	2012	Retro	International	Yes	NR	185	NR	NR	80	NR	48–160	NR	Not specified
Lin	2016	Single	NR	NR	Retro	Australia	Yes	Aquilion One	NR	65	40	80	Variable	160	MIStar	Standard and delay corrected svd
Yu	2016	Single	2011	2015	Retro	China	Yes	Siemens Somatom	NR	74.5	60	80	Variable	100	MIStar	Ddsvd
Bivard	2017	Multicenter	2012	2016	Retro	International	NR	Somatom/Aquilion One	NR	45–60	40	NR	NR	41–160	MIStar	Ddsvd
Copen	2017	Single	NR	NR	Retro	United States	Yes	LightSpeed	NR	66	40–45	NR	3	80	CT Perfusion 4D	Delay corrected svd
Chen	2019	Single	2005	2010	Retro	Australia	NR	Phillips/Aquilion One	189	60	NR	NR	NR	48–160	MIStar	Delay-sensitive ssvd and ddsvd
Qiu	2019	Multicenter	2010	2014	Retro	International	NR	NR	168	NR	NR	NR	NR	>80	CT Perfusion 4D	Delay-insensitive deconvolution
Laredo	2020	Single	2010	2017	Retro	Spain	NR	Siemens Somatom	175	39	50	80	1.5	98	MIStar	Ddsvd

NR, not recorded; Bivard^a^, Cerebrovascular Diseases, 2011; Bivard^b^, Brain, 2011; sSVD, standard singular value decomposition; ddSVD, delay and dispersion corrected singular value decomposition; ^**+**^mean.

**Table 2:** Assessment of risk of bias of studies using the QUADAS-2 tool.

Study	Risk of bias								Applicability concerns					
	Patient selection		Index test		Reference standard		Flow and timing		Patient selection		Index test		Reference standard	
Schaefer, 2006	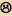	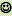	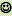	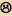	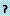	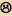	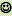	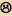	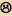	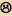	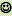	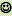	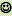	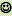
Murphy, 2006	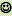	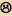	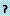	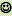	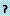	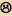	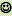	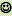	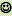	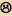	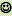	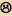	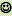	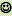
Wintermark, 2006	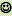	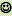	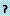	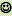	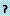	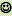	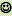	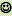	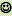	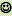	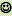	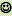	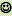	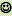
Murphy, 2008	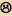	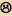	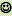	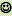	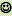	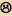	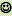	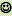	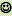	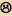	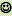	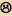	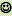	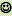
Bivard^a^, 2011	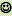	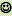	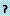	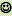	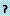	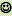	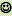	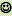	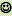	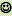	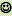	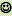	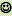	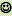
Bivard^b^, 2011	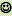	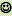	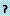	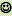	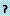	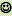	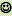	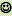	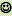	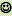	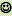	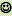	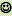	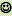
Campbell, 2011	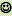	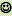	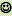	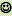	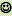	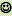	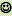	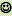	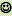	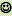	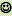	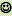	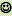	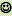
Kamalian, 2011	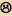	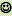	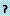	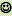	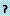	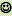	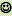	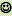	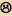	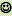	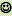	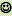	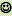	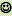
Payabvash, 2011	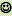	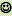	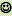	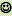	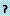	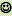	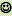	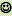	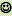	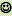	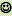	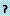	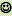	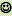
Campbell, 2012	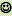	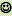	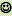	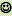	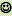	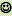	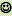	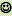	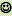	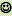	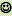	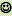	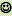	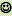
Kamalian, 2012	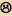	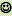	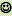	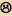	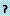	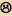	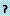	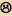	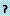	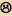	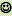	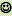	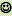	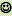
Bivard, 2013	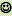	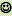	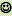	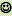	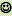	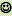	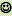	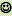	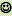	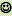	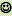	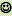	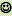	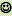
Bivard, 2014	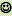	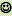	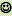	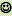	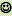	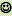	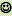	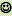	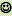	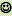	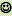	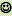	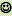	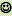
Eilaghi, 2014	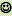	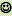	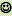	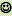	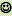	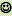	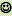	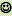	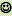	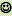	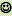	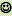	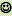	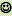
McVerry, 2014	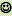	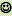	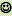	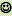	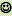	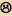	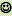	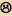	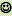	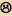	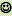	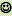	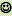	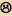
D’Esterre, 2015	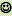	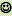	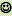	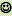	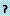	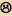	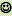	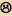	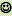	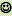	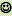	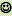	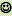	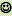
Cereda, 2016	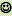	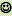	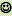	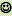	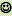	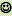	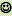	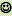	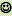	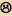	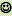	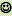	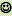	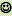
Yu, 2016	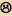	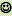	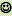	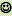	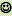	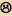	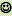	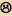	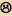	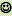	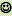	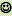	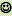	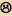
Lin, 2016	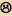	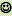	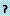	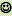	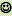	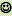	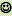	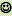	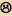	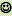	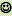	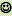	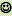	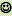
Bivard, 2017	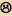	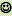	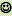	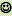	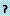	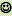	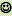	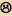	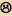	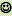	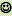	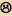	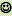	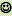
Copen, 2017	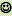	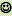	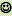	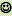	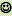	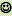	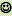	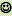	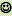	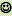	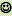	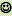	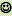	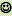
Chen, 2019	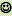	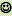	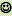	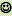	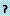	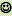	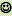	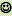	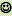	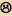	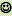	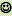	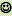	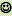
Qiu, 2019	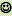	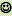	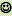	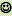	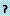	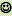	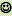	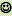	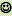	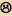	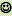	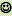	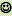	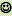
Laredo, 2020	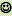	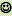	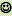	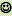	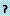	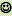	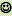	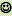	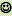	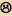	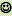	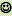	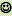	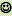

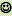
Low risk 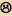
High risk 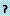 Unclear risk.

### Study quality

The results of the QUADAS-2 tool (17) are shown in Table 2. Only six studies were classified by both reviewers as having an overall judgment of low risk of bias and low concern regarding applicability (19, 20, 43–46). Only seven studies reported blinding of assessors to analysis of perfusion imaging and the reference test (15, 23, 25, 26, 43, 44, 46). Two studies reported adherence to STARD (52) (Standards for Reporting of Diagnostic Accuracy) guidelines^1^ (43, 48). One study included a flow diagram outlining patient selection process (23). Others included details of excluded patients in the Results section (15, 16, 18–22, 24–26, 42, 45, 46, 49) or in online data supplement (47).

### Study design

#### Brain coverage, scan acquisition time, and post-processing algorithms

Reported brain coverage, scan acquisition time, and post-processing methods varied between studies (see Table 1). In general, three main deconvolution methods were used. Nine studies used delay and dispersion correction deconvolution models producing delay time (DT) maps with the post-processing software, MIStar (15, 16, 18–22, 48, 49). Six studies used standard or ‘delay sensitive’ (16, 23, 27, 28, 43, 44) deconvolution which does not correct for arrival delay of contrast and six studies used ‘delay-insensitive’ also known as ‘delay corrected’ which does correct for arrival delay of contrast (23, 28, 42, 45–47). Studies investigating the impact of post-processing methods showed thresholds to define core and penumbra varied depending on the deconvolution method used (16, 20, 27, 28).

#### Time to reference standard

Imaging reference was a combination of MRI and NCT (6 studies) (15, 28, 42, 47, 49, 50), MRI alone (16 studies) (16, 18–23, 26, 27, 41, 44–46, 48, 51), or NCT alone (2 studies) (24, 25). Two studies investigating thresholds for penumbra also used contemporaneous MR-PWI (43) or CTP or MRP at 24 h (15). Time interval to reference varied between study protocols. For studies investigating core, most did not record median or mean time to reference imaging. When reported, median interval to acute MRI was 27.5 (IQR 22–31.5) min (see Table 1; Supplementary material) (16, 21, 22, 26, 27, 41, 44, 46). Other studies used DWI 24 h after CTP in patients with major reperfusion (18–23), delayed MRI, at a median interval of 38 h in patients with complete recanalization (48) or at 5–7 days (45) to determine infarct core. For studies investigating penumbra, several studies did not report median or mean time to reference scan (see Table 2 in Supplementary material).

#### Vessel occlusion and reperfusion therapies

Seventeen studies included patients with evidence of large vessel occlusion on imaging using CTA, MRA, or DSA (16, 18, 23–25, 27, 28, 41–45, 47–51). When reported, recanalization was determined using three scales: the Mori (50), thrombolysis in myocardial infarction (TIMI) (23, 49), and modified thrombolysis in cerebral infarction (mTICI) scales (18, 42, 48). Reperfusion therapies included intravenous thrombolysis (15, 16, 18–26, 41, 43–46, 50), local intra-arterial fibrinolysis (24, 25, 50), and clot retrieval in combination with thrombolysis (16, 18, 26, 50). Since 2015, three studies examined perfusion parameters following treatment with endovascular clot retrieval alone (42, 47, 48).

#### Imaging analysis

Imaging analysis performed by studies included either a region of interest analysis (24, 25, 42–44, 47, 48, 50), or as defined by the authors a pixel (18–23, 41, 46, 49) or voxel-based analysis (15, 16, 26–28, 45, 51). The term ‘voxel’ is used to encompass both pixels and voxels. Eleven studies used semi-automatic or automatic delineation of infarct on MRI using signal intensity thresholds (16, 18–23, 27, 28, 46, 48). Others manually outlined the maximal visual extent of the infarct on follow-up imaging (MRI or NCT) (15, 24–26, 41–44, 47, 49–51). Voxels within both the hypoperfusion region on CTP and infarct on DWI or NCT were regarded as “true-positive,” and those within the region of hypoperfusion on the index test but not in the reference test was “false-positive.” Most studies analyzed only the ischemic hemisphere (15, 16, 18–23, 43, 44). One study delineated non-infarcted voxels in the whole brain parenchyma (41). Two studies only examined the section with the largest tissue which evolved to infarction (27, 28).

Two different approaches were employed to determine the ability of perfusion parameters to accurately identify the final infarct on the follow-up scan. The first, a volume analysis approach defined the optimal parameter as the one with the least volume difference between index and reference standard. The second used receiver operator characteristic (ROC) curve analysis to identify parameters with the highest sensitivity and specificity to predict the final infarction on the reference image. Most diagnostic accuracy studies used ROC curve analysis alone (27, 28, 41, 42, 45, 47, 50, 51) or in conjunction with a volume analysis approach (15, 16, 18–22, 24–26, 44, 46, 49). Only one study used a volume analysis approach alone (48). Sensitivity, specificity, area under the curve (AUC), and the Youden index for the optimal parameters for core and penumbra from each study are included in Tables 3 4, respectively. The parameter with the highest Youden index of 0.94 was a product of CBF and CBV which at a threshold of 31.3 differentiated core from penumbra in an early study (24). The Youden index for rCBF <31% to represent ischemic core was less than 0.5 in three out of five studies (median 0.60, IQR 0.48, 0.63) (15, 16, 44).

**Table 3:** Parameters and thresholds for identifying core.

Authors	Year	Median onset time to CTP (min)	Image analysis	Statistical analysis	Parameter	Threshold	Units	Sensitivity	Specificity	AUC	Youden index
Schaefer	2006	144	ROI	ROC	CBF ratio	<0.32	NR	NR	NR	NR	NR
Murphy	2006	133**	ROI	ROC/Volume	CBF.CBV	<31.3	NR	0.97	0.97	NR	0.94
Wintermark	2006	NR	Voxel	ROC	aCBV	2	mL X 100 g^−1^	NR	NR	0.93	NR
Murphy	2008	133**	ROI	ROC/Volume	CBF.CBV	<8.14	NR	0.95	0.94	NR	0.89
Bivard^a^	2011	195	Voxel	ROC/Volume	rCBF	45	%	NR	NR	0.79	NR
Bivard^b^	2011	NR	Voxel	ROC/Volume	rCBF (DT)	40 (>2)	%	0.93	0.78	0.86	0.71
Campbell	2011	190	ROI	ROC/Volume	rCBF	31	%	0.72	0.73^€^	0.79	0.45
								0.72	0.88^§^	0.79	0.6
Kamalian	2011	246	Voxel	ROC	rCBF	16	%	0.8	0.83	0.88	0.63
Payabvash	2011	222^**+**^	Voxel	ROC	rCBF	0.42^$^	%	0.54	0.80	0.72	0.34
						0.16	%	0.54	0.79	0.73	0.33
Campbell	2012	213^+^	ROI	Volume	rCBF (TTP)	31 (>4)	%	NR	NR	NR	NR
Bivard	2013	NR	Voxel	ROC/Volume	rCBF (DT)	40 (>2)	%	0.73	0.93	0.86	0.66
Bivard	2014	NR	Voxel	ROC/Volume	rCBF	50	%	0.66	0.81	0.75	0.47
Eilaghi	2014	NR	Voxel	ROC	rCBF	0.78	NR	0.89	0.93	NR	0.82
McVerry	2014	196	Voxel	ROC/Volume	rCBF	45	%	NR	NR	NR	NR
d’Esterre	2015	NR	ROI	ROC	Tmax^**#**^	16.2	s	0.85	0.83	0.91	0.68
		NR			aCBF^**^**^	9.5	mL/100 g/min	0.84	0.86	0.88	0.7
Cereda	2016	185	Voxel	ROC/Volume	rCBF	38	%	0.67	0.87	NR	0.54
Lin	2016	174	Voxel	ROC/Volume	rCBF (DT)	30	%	0.8	0.91	NR	0.71
Yu	2016	NR	Voxel	ROC/Volume	rCBF (DT)	30 (>3)	%	0.64	0.76	0.76	0.4
Bivard	2017	NR	Voxel	ROC/Volume	rCBF	30	%	0.84	0.77	0.83	0.61
		NR				20	%	0.91	0.87	0.89	0.78
Copen	2017	NR	Voxel	ROC/Volume	rCBF	29	%	0.79	0.85	0.89	0.64
Chen	2019	NR	Voxel	ROC/Volume	rCBF	30	%	0.6	0.88	0.74	0.48
Qiu	2019	92	ROI	ROC	Tmax^**#**^	15.7	s	0.81	0.87	0.93	0.68
		244			aCBF^**^**^	9.2	mL/100 g/min	0.77	0.82	0.9	0.59
Laredo	2020	81	ROI	Volume	rCBF	25	%	NR	NR	NR	NR
		269				30	%	NR	NR	NR	NR

NR, not recorded; Bivard^a^, Cerebrovascular Diseases, 2011; Bivard^b^, Brain, 2011; **average; *median; ^**+**^mean; ROI, region of interest; ROC, receiver operating characteristic; AUC, area under curve; ^#^onset to sca*n* < 180 min; ^**^**^onset to sca*n* > 180 min; ^€^within perfusion lesion; ^§^whole brain; ^$^highly vulnerable regions.

**Table 4:** Parameters and thresholds for identifying penumbra.

Authors	Year	Image analysis	Statistical analysis	Parameter	Threshold	Units	Sensitivity	Specificity	AUC	Youden index
Schaefer	2006	ROI	ROC	CBF ratio	>0.44	NR	NR	NR	NR	NR
Murphy	2006	ROI	ROC/Volume	CBF.CBV	>31.3	NR	0.97	0.97	NR	0.94
Wintermark	2006	Voxel	ROC	rMTT	145	%	NR	NR	0.96	NR
Murphy	2008	ROI	ROC/Volume	CBF.CBV	>8.14	NR	0.95	0.94	NR	0.89
Bivard^b^	2011	Voxel	ROC/Volume	rDT	2	s	0.82	0.9	0.86	0.72
Campbell	2012	ROI	ROC/Volume	Tmax	6	s	0.91	0.7	0.87	0.61
Kamalian	2012	Voxel	ROC	rMTT	249	%	0.65	0.8	0.78	0.45
Bivard	2013	Voxel	ROC/Volume	DT	2	s	0.83	0.82	0.86	0.65
Bivard	2014	Voxel	ROC/Volume	rTTP	5	s	0.72	0.82	0.79	0.54
McVerry	2014	Voxel	ROC/Volume	DT	2	s	NR	NR	NR	NR
Yu	2016	Voxel	ROC/Volume	DT	3	s	0.75	0.74	0.81	0.49
Lin	2016	Voxel	ROC/Volume	Tmax	6	s	0.80	0.91	NR	0.71
Chen	2019	Voxel	ROC/Volume	Tmax	6	s	0.72	0.84	0.78	0.56

NR, not recorded; Bivard^b^, Brain, 2011; ROI, region of interest; ROC, receiver operating characteristic; AUC, area under curve. The maximum value of the Youden index is 1 and a Youden index of less than 0.5 does not meet the optimal performance standard for a diagnostic test.

### Perfusion parameters and thresholds

#### Parameters for core

**Figure 2:**
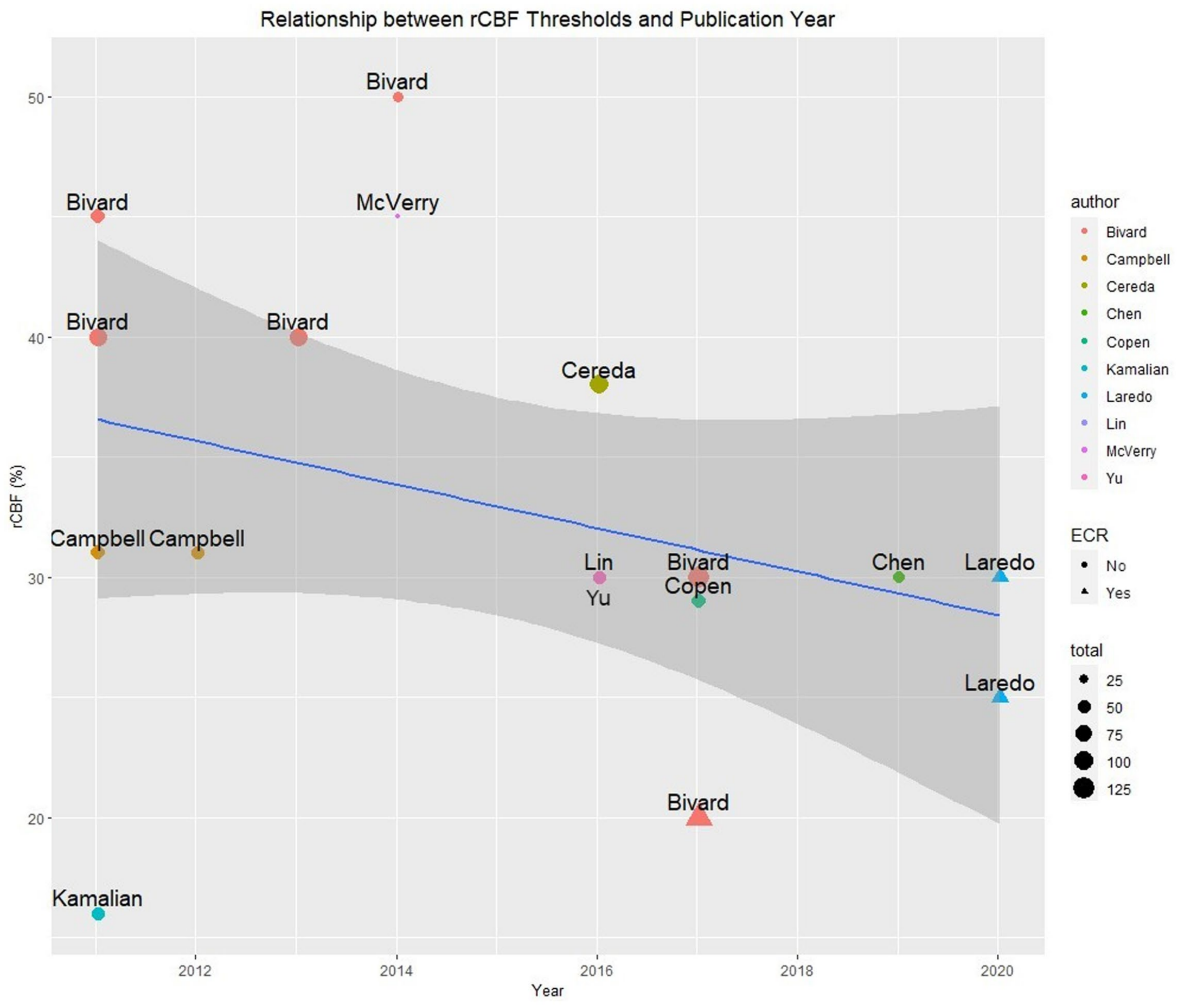
Reported optimal rCBF (%) thresholds to identify core by publication year, sample sizes, author, and reperfusion therapy (triangle—ECR only).

The most reported parameter was rCBF, described in a total of 16 studies. The median rCBF threshold was <30% (IQR 30–40%). Figure 2 shows that rCBF thresholds varied between studies even from the same research groups. A regression analysis evaluating the relationship between published rCBF thresholds over a 10-year period showed no statistically significant association (regression coefficient: 0.1; *p* = 0.21) between rCBF threshold values and the publication year (see Figure 2). Five studies constrained rCBF within the perfusion lesion (using TTP > 4 s or DT > 2 s or DT > 3 s) to reduce false positives from regions of leukoaraiosis (15, 20, 22, 23, 43). In the early time window, one study showed thresholds were dependent on the reperfusion method used. They reported a lower threshold of rCBF <20% in patients treated with clot retrieval due to faster reperfusion [see Figure 2; Bivard (18)].

The definition of ‘infarct core’ continued to vary between studies (8, 9), with some defining core using the acute DWI lesion (16, 21, 22, 26, 27, 41, 43, 44, 46, 51), and others using delayed imaging in patients with complete recanalization [TIMI 3 (23, 49), TICI 2b (42, 47), or TICI 3 (42, 47, 48)] and clinical improvement. Five studies defined core in patients with evidence of ‘major’ reperfusion following treatment with thrombolysis or clot retrieval. This was defined as more than 80% reduction in the perfusion lesion volume between acute CTP and 24-h MR imaging, using MTT >145% of the normal tissue (16, 19, 20, 22) or Tmax >6 s (15). Others did not specify the threshold for MTT (21). One study used TICI 3 recanalization and ‘complete’ reperfusion defined as more than 90% reduction from baseline CTP using DT > 3 s (18). One early study defined core as the infarcted tissue on delayed NCT or T2-weighted MRI with reduced CBV and CBF on initial CTP (50). Another early study defined penumbra as the tissue with CBF <25 mL/100 g/min, which did not infarct on delayed NCT and then applied logistic regression to differentiate between penumbra and core in the gray (24) and white (25) matter in recanalized patients.

#### Parameters for penumbra

Three studies reported Tmax >6 s as the most accurate parameter for penumbra (AUC 0.78–0.87) (16, 23, 43). Using the MIStar software, three studies found DT > 2 s was the most accurate parameter and threshold for penumbra (20, 22, 49). One study (*n* = 22) reported a higher threshold (DT > 3 s) in a Chinese population (15). Two older studies suggested that relative MTT (28, 41) with thresholds varying from 145 to 249% was optimal for penumbra.

Three different methods were used to define penumbra. One pivotal study (43) used MR-PWI within an hour of CTP and matched thresholds for penumbra to MR-PWI threshold of Tmax >6 s. Five studies defined penumbra using CTP parameters and thresholds to identify ‘no or minimal reperfusion’ (<20% reperfusion) at 24 h (15, 16, 19, 20, 22). Studies defined minimal or no reperfusion using MTT >145% of the normal tissue (16, 19, 20, 22) or Tmax>6 s (15). Three studies used infarct growth between the area of hypoperfusion on CTP and the infarcted region found on 24-h MRI (23, 41) alone or combined with delayed NCT (49) in patients with persistent symptomatic occlusion on follow-up CTA or MRA (23, 41, 49). One study assumed persistent occlusion by including patients with vessel occlusion who in the absence of thrombolysis had no late clinical or radiological confirmation of reperfusion (28). Only two studies investigated thresholds for differentiating penumbra or ‘tissue at risk’ from ‘oligemia.’ (28, 50) Oligemia or ‘penumbra that recovers’ was defined as hypoperfusion on CTP (28) or reduced CBF and normal CBV (50), which did not infarct on delayed imaging.

#### Perfusion parameters and time

The relationship between thresholds and time to reperfusion was investigated in three studies (42, 47, 48). Two of these studies demonstrated a statistically significant relationship between Tmax and aCBF and time to reperfusion from CTP (42, 47). If reperfusion occurred within 90 min, the optimal Tmax thresholds for the core was higher, and aCBF threshold lower than if reperfusion occurred between 90 and 180 min (42, 47). Optimal threshold values reported for rCBF were also lower if reperfusion occurred within 90 min (42) or when recanalization occurred within 4.5 h of stroke onset (see Figure 2; Laredo 2020) (48).

#### Thresholds for gray and white matter

Eight studies investigated thresholds for the gray matter (GM) and the white matter (WM) (16, 24, 25, 28, 42, 45, 47, 50). Two early studies reported higher threshold values for the product of CBF and CBV in GM compared to WM (24, 25). For identifying penumbra, studies reported higher thresholds in WM than GM for aMTT (*n* = 23) (28) and Tmax (*n* = 31) (16) and lower thresholds in WM than GM for parameters, aCBF, and aCBV (*n* = 14) (50). However, thresholds for DT were constant (*n* = 31) (16). For identifying core, no significant difference in Tmax thresholds was reported between GM and WM (42, 47). Two studies reported consistently lower thresholds in WM than GM for CBF (16, 47), but one study (*n* = 132) did not (42). Reported thresholds for CBV were also generally lower in WM than GM (42, 47). One study reported higher thresholds for rCBF in highly vulnerable brain regions including the caudate body, putamen, and insular ribbon (51).

## Discussion

An important finding of this systematic review is that a single parameter and threshold may not always accurately differentiate penumbra from core and oligemia. Although each diagnostic accuracy study has reported an optimal parameter and threshold for core and penumbra as the gold standard, these varied significantly depending on the cohorts investigated. This is especially evident from the studies published by the same research groups which reported different parameters for penumbra (15, 19, 22, 28, 43, 50) and varying rCBF thresholds for core (16, 18–23, 27, 46, 50) (see Figure 2).

In the assessment of a diagnostic test, the results obtained are usually evaluated in an independent cohort of patients to ensure replicability and reliability. Only two studies validated reported thresholds in an independent dataset to confirm their findings (45, 49). Most studies that used small sample sizes did not perform cross-validation or follow the STARD (52) guidelines and continued to be limited by non-blinding to the reference standard. Consequently, the results varied even when published by the same group. The use of time-dependent thresholds for ischemic core, such as rCBF and Tmax, which are impacted by time to reperfusion, and highly effective reperfusion therapies such as thrombectomy may also impact variability (18, 47, 48) (8, 10). Additionally, 15 studies defined a single threshold for all brain regions irrespective of collaterals and the gray and white matter. Differing thresholds seem to exist between the gray and white matter for some parameters (16, 28, 42, 47, 50) and brain regions (51), but further exploration of these findings is needed. Developing maps that overcome the issue of time-dependent thresholds and applying different thresholds for the gray and white matter or brain regions may increase accuracy in determining core infarct volume.

As previously reported, significant heterogeneity in technical imaging parameters including acquisition times, varied pre- and post-processing techniques (15, 28, 53), scan duration, modality and timing of reference standard, time from stroke onset to CTP scan, and multiple definitions for penumbra and core may also contribute to the variability in reported thresholds and parameters. Additionally, patient factors (10) including the presence of white matter disease, collaterals, location of stroke (anterior or posterior), cardiac output, vessel occlusion or stenosis, and hematocrit ratio may also be important. Comparable to studies investigating MR perfusion, most CT perfusion studies were unable to distinguish the penumbral tissue from the benign oligemia and may have overestimated the region of hypoperfusion representative of the penumbra (28, 54).

We have documented sensitivity, specificity, and AUC as reported by the published journal articles or their supplementary pages (see Tables 3, 4). In several cases, the reported values for AUC seem to be dissociated with values for sensitivity and specificity (15, 18, 20, 27, 42, 44, 46, 47, 51). This situation can arise as the AUC is calculated for the range of thresholds for a test, and the sensitivity and specificity of a test are described for a particular threshold. The Youden index was also used to define the performance at the optimal cutoff point. Using the rCBF threshold of 31% to describe the infarct core, the median value of the Youden index was 0.6. In three of the five studies, the calculated Youden index was less than optimal (15).

### Study limitations

There are several strengths of this systematic review. First, we followed the guidance outlined by the Cochrane Database of Systematic Reviews and PRISMA-DTA guidelines. Second, we performed an extensive literature search using different electronic databases with two review authors conducting data extraction. Third, the assessment of quality was carried out by two independent review authors using the QUADAS-2 tool (17) for quality assessments. While the QUADAS-2 tool (17) had suggested that 14 studies were classified as high quality, these studies did not fully meet the STARD (52) guidelines (described above). Finally, we only included studies that used co-registration of index images to the reference standard to ensure the accuracy of tissue location and volume analysis.

The following limitations are worth noting. We only included studies published in the English language, and most included studies were retrospective cohort studies, with sample sizes of less than 50 patients which can run the risk of producing an overestimation of sensitivity and specificity. We acknowledge that a limitation of the Youden index is its inability to distinguish a test with a high sensitivity and low specificity from one with low sensitivity and high specificity. Studies that were published from the same study sites and groups may have included the same patients more than once. Despite approaching authors (18–23), we were unable to obtain this information. Only seven studies were blinded which increases the risk of bias in interpretation of the index test and reference results. Due to our selection criteria, we have reported results pertaining to anterior circulation stroke from mostly two post-processing software manufacturers, excluding studies comparing other commonly used post-processing software such as RAPID® (29–31), Vitrea (55), and Sphere (56). Finally, there was significant methodological heterogeneity between studies which unfortunately precluded further analysis.

## Conclusion

Although CT perfusion has found its place in clinical practice, clinicians involved in the hyperacute care of stroke patients need to recognize its current limitations. An accurate determination of infarct volume is essential to ensure the appropriate selection of patients for reperfusion therapies and identify patients for transfer to regional stroke centers. This may not always be possible with a single threshold and parameter. Prospectively designed, multicenter diagnostic accuracy studies following STARD (52) guidelines using standardized imaging acquisition and analysis protocols are still needed to improve the accuracy of CT perfusion. Future research may involve thresholds that consider variations in the gray and white matter and identifying parameters that can precisely differentiate the penumbra from the oligemic tissue and core from the penumbra irrespective of time from stroke onset. The creation of voxel maps using the product of rCBF, rCBV, and hypoattenuation in Hounsfield units on NCT may enable a less time-dependent and more accurate determination of the core.

## Data availability statement

The original contributions presented in the study are included in the article/Supplementary material, further inquiries can be directed to the corresponding author.

## Author contributions

TT: Writing – original draft, Writing – review & editing, Formal analysis. SA: Formal analysis, Writing – review & editing. AL: Investigation, Writing – review & editing, Formal analysis. CD: Investigation, Writing – review & editing, Formal analysis. HM: Conceptualization, Writing – review & editing. TP: Methodology, Writing – review & editing, Conceptualization.

## Footnotes

^1^ http://www.stard-statement.org/

## Abbreviations

CBF: Cerebral blood flow
CBV: Cerebral blood volume
CTP: Computed tomography perfusion
DT: Delay time
MRI: Magnetic resonance imaging
MRI-PWI: Magnetic resonance perfusion-weighted imaging
MTT: Mean transit time
mTICI: Modified thrombolysis in cerebral infarction
NCT: Non-contrast computed tomography
PET: Positron emission tomography
Tmax: Time to maximum
TIMI: Thrombolysis in myocardial infarction
