# Supplementary material for: Assessing the diagnostic accuracy of CT perfusion: a systematic review
Source: Front Neurol. 2023 Oct 11;14:1255526. doi: 10.3389/fneur.2023.1255526 (PMC10598661; doi:10.3389/fneur.2023.1255526)
Supplement: Supplementary file 1 [file Table_1.docx]

Table 1 – Study characteristics investigating core

| **Authors** | **Year** | **Total** | **Mean Age** | **Male (%)** | **Median NIHSS** | **Median Onset Time to CTP (mins)** | **Imaging Reference** | **Median Time from CTP to Ref** | **Vessel Occlusion** | **Reperfusion Therapies** | **Median Onset to reperfusion (mins)** | **Parameter** | **Threshold** | **Units** |
| --- | --- | --- | --- | --- | --- | --- | --- | --- | --- | --- | --- | --- | --- | --- |
| Schaefer | 2006 | 14 | 70 | 29 | 17 | 144 | MRI/NCT | 3.5 days | Y | iv or ia tpa/ECR | NR | CBF ratio | <0.32 | NR |
| Murphy | 2006 | 16 | 63.8** | 40 | 13** | 133** | NCT | NR | Y | iv or ia tpa | NR | CBF.CBV | <31.3 | NR |
| Wintermark | 2006 | 25 | 63 | 58 | 15.3**^+^** | NR | MRI | 25 mins | Y | tpa | NR | aCBV | 2 | mL X 100g^-1^ |
| Murphy | 2008 | 16 | 63.8** | 40 | 13** | 133** | NCT | NR | Y | iv or ia tpa | NR | CBF.CBV | <8.14 | NR |
| Bivard^a^ | 2011 | 57 | 74 | NR | 16 | 195 | MRI | 28 mins | NR | tpa | NR | rCBF | 45 | % |
| Bivard^b^ | 2011 | 67 | 70 | NR | 13 | NR | MRI | 19 mins | NR | tpa | NR | rCBF(DT) | 40(>2) | % |
| Campbell | 2011 | 54 | 71.8 | 48 | NR | 190 | MRI | 27 mins | Y | tpa | NR | rCBF | 31 | % |
| Kamalian | 2011 | 48 | 71.6 | 46 | 13 | 246 | MRI | 34 mins | Y | NR | NR | rCBF | 16 | % |
| Payabvash | 2011 | 90 | NR | 60 | NR | 222**^+^** | MRI | 24 mins**^+^** | Y | NR | NR | rCBF | 0.42^$^ | % |
|  |  |  |  |  |  |  |  |  |  |  |  | rCBF | 0.16 | % |
| Campbell | 2012 | 49 | 70.4 | 45 | 16.5 | 213**^+^** | MRI | 31 mins**^+^** | Y | tpa | NR | rCBF(TTP) | 31(>4) | % |
| Bivard | 2013 | 67 | 70 | NR | 13 | NR | MRI | NR | NR | tpa | NR | rCBF(DT) | 40(>2) | % |
| Bivard | 2014 | 33 | 72 | NR | 12 | NR | MRI | NR | NR | tpa | NR | rCBF | 50 | % |
| Eilaghi | 2014 | 203 | NR | NR | NR | NR | MRI | NR | Y | tpa | NR | rCBF | 0.78 | NR |
| McVerry | 2014 | 16 | 72 | NR | 15 | 196 | MRI/NCT | 29 hrs | Y | NR | NR | rCBF | 45 | % |
| d'Esterre**^§^** | 2015 | 42 | 66.9 | 46.3 | 17 | 171.3**^+^** | MRI/NCT | NR | Y | ECR | 65.5**^+^** | Tmax**^#^** | 16.2 | secs |
|  |  |  |  |  |  |  |  |  |  |  |  | aCBF**^^^** | 9.5 | mL/100g/min |
| d'Esterre**^€^** | 2015 | 42 | 69.6 | 38.1 | 17 | 159.2**^+^** | MRI/NCT | NR | Y | ECR | 133.5**^+^** | Tmax**^#^** | 12.4 | secs |
|  |  |  |  |  |  |  |  |  |  |  |  | Tmax**^^^** | 10.3 | secs |
| Cereda | 2016 | 103 | 68 | NR | 16 | 185 | MRI | 36 mins | NR | tpa/ECR | NR | rCBF | 38 | % |
| Lin | 2016 | 44 | 74 | NR | 14 | 174 | MRI | NR | Y | tpa | NR | rCBF(DT) | 30(>3) | % |
| Yu | 2016 | 47 | 70 | 45 | 11 | NR | MRI/NCT | NR | NR | tpa | NR | rCBF(DT) | 30(>3) | % |
| Bivard | 2017 | 132 | 63 | 42 | 13 | NR | MRI | NR | Y | tpa | NR | rCBF | 30 | % |
| Bivard | 2017 | 132 | 65 | 48 | 15 | NR | MRI | NR | Y | ECR | 239 | rCBF | 20 | % |
| Copen | 2017 | 58 | 69.2 | 60 | 8 | NR | MRI | 19.5 mins | NR | tpa | NR | rCBF | 29 | % |
| Chen | 2019 | 40 | 74* | NR | 16 | NR | MRI | 29 mins | Y | tpa/ECR | NR | rCBF | 30 | % |
| Qiu**^#^** | 2019 | 54 | 71 | 48 | 17 | 92 | MRI/NCT | NR | Y | ECR | 112 | Tmax | 15.7 | secs |
| Qiu**^^^** | 2019 | 83 | 62 | 42 | 17 | 244 | MRI/NCT | NR | Y | ECR | 86 | aCBF | 9.2 | mL/100g/min |
| Laredo | 2020 | 54 | 73* | 52 | 16 | 81 | MRI | 38 hrs | Y | ECR | 195 | rCBF | 25 | % |
| Laredo | 2020 | 50 | 76* | 42 | 17 | 269 | MRI | 38 hrs | Y | ECR | 379 | rCBF | 30 | % |

NR = Not recorded; Bivard^a^ = Cerebrovascular Diseases, 2011; Bivard^b^ = Brain, 2011; **average; *median; **^+^**mean; tpa = thrombolysis; ECR= clot retrieval; **^§^**CTP to reperfusion<90min; **^€^**CTP to reperfusion 90-180min; ^#^onset to scan<180min; **^^^**onset to scan>180min; ^$^highly vulnerable regions

Table 2 – Study characteristics investigating penumbra

| **Authors** | **Year** | **Total** | **Mean Age** | **Male (%)** | **Median NIHSS** | **Median Onset to CTP (mins)** | **Imaging Reference** | **Median Time from CTP to Ref** | **Vessel Occlusion** | **Reperfusion Therapies** | **Parameter** | **Threshold** | **Units** |
| --- | --- | --- | --- | --- | --- | --- | --- | --- | --- | --- | --- | --- | --- |
| Schaefer | 2006 | 14 | 70 | 29 | 17 | 144 | MRI/NCT | 3.5 days | Y | iv or ia tpa or ECR | CBF ratio | >0.44 | NR |
| Murphy | 2006 | 16 | 63.8** | 40 | 13** | 133** | NCT | NR | Y | iv or ia tpa | CBF.CBV | >31.3 | NR |
| Wintermark | 2006 | 46 | 63 | 58 | 15.3** | NR | MRI | 3.5 days | Y | tpa | rMTT | 145 | % |
| Murphy | 2008 | 16 | 63.8** | 40 | 13** | 133** | NCT | NR | Y | iv or ia tpa | CBF.CBV | >8.14 | NR |
| Bivard^b^ | 2011 | 124 | 70 | NR | 13 | NR | MRI | 24 hrs | NR | tpa | rDT | 2 | secs |
| Campbell | 2012 | 49 | 70.4 | 45 | 16.5 | 213**^+^** | MRP | 31 mins**^+^** | Y | tpa | Tmax | 6 | secs |
| Kamalian | 2012 | 23 | 79 | 35 | 13 | 252 | MRI/NCT | 2 days | Y | None | rMTT | 249 | % |
| Bivard | 2013 | 146 | 70 | NR | 13 | NR | MRI | NR | NR | tpa | DT | 2 | secs |
| Bivard | 2014 | 67 | 72 | NR | 12 | NR | MRI | NR | NR | tpa | rTTP | 5 | secs |
| McVerry | 2014 | 19 | 71 | NR | 18 | 164 | MRI/NCT | 26 hrs | Y | NR | DT | 2 | secs |
| Lin | 2016 | 40 | 72 | NR | 17 | 141 | MRI | NR | Y | tpa | Tmax | 6 | secs |
| Yu | 2016 | 22 | 74 | 70 | 13 | NR | MRI/NCT | NR | NR | tpa | DT | 3 | secs |
| Chen | 2019 | 31 | 74* | NR | 16 | 189 | MRI | 29 mins | Y | tpa | Tmax | 6 | secs |

NR = Not recorded; Bivard^b^ = Brain, 2011; **average; *median; **^+^**mean; ROC = receiver operating characteristic; ROI = region of interest; tpa = thrombolysis; ECR= clot retrieval; mCBF= mean CBF

Table 3 – Excluded studies

| **Excluded Studies** | **Reason for Exclusion** |
| --- | --- |
| Biesbroek JM, Niesten JM, Dankbaar JW, Biessels GJ, Velthuis BK, Reitsma JB, et al. Diagnostic accuracy of ct perfusion imaging for detecting acute ischemic stroke: A systematic review and meta-analysis. *Cerebrovasc Dis*. 2013;35:493-501 | Meta-analysis |
| Dani KA, Thomas RG, Chappell FM, Shuler K, MacLeod MJ, Muir KW, et al. Computed tomography and magnetic resonance perfusion imaging in ischemic stroke: Definitions and thresholds. *Ann Neurol*. 2011;70:384-401 | Systematic Review |
| Xin Y, Han FG. Diagnostic accuracy of computed tomography perfusion in patients with acute stroke: A meta-analysis. *J Neurol Sci*. 2016;360:125-130 | Meta-analysis |
| Bandera E, Botteri M, Minelli C, Sutton A, Abrams KR, Latronico N. Cerebral blood flow threshold of ischemic penumbra and infarct core in acute ischemic stroke: A systematic review. *Stroke*. 2006;37:1334-1339 | Systematic Review |
| Lim N, E, Chia B, Bulsara M, K, Parsons M, Hankey G, J, Bivard A: Automated CT Perfusion Detection of the Acute Infarct Core in Ischemic Stroke: A Systematic Review and Meta-Analysis. Cerebrovasc Dis 2023;52:97-109. doi: 10.1159/000524916 | Systematic Review |
| Amukotuwa S, Straka M, Aksoy D, Fischbein N, Desmond P, Albers G, et al. Cerebral blood flow predicts the infarct core: New insights from contemporaneous diffusion and perfusion imaging. *Stroke*. 2019;50:2783-2789 | Included patients who had MRP as index test |
| Klotz E, König M. Perfusion measurements of the brain: Using dynamic ct for the quantitative assessment of cerebral ischemia in acute stroke. *Eur J Radiol*. 1999;30:170-184 | Does not co-register images |
| Bao DZ, Bao HY, Yao LZ, Pan YG, Zhu XR, Yang XS, et al. 64-slice spiral ct perfusion combined with vascular imaging of acute ischemic stroke for assessment of infarct core and penumbra. *Experimental and Therapeutic Medicine*. 2013;6:133-139 | Does not investigate multiple thresholds |
| Parsons MW, Pepper EM, Bateman GA, Wang Y, Levi CR. Identification of the penumbra and infarct core on hyperacute noncontrast and perfusion ct. *Neurology*. 2007;68:730-736 | Does not investigate multiple thresholds |
| Rai AT, Carpenter JS, Peykanu JA, Popovich T, Hobbs GR, Riggs JE. The role of ct perfusion imaging in acute stroke diagnosis: A large single-center experience. *J Emerg Med*. 2008;35:287-292 | Does not investigate multiple thresholds |
| Ho CY, Hussain S, Alam T, Ahmad I, Wu IC, O'Neill DP. Accuracy of ct cerebral perfusion in predicting infarct in the emergency department: Lesion characterization on ct perfusion based on commercially available software. *Emerg Radiol*. 2013;20:203-212 | Does not investigate multiple thresholds |
| Huisa BN, Neil WP, Schrader R, Maya M, Pereira B, Bruce NT, et al. Clinical use of computed tomographic perfusion for the diagnosis and prediction of lesion growth in acute ischemic stroke. *J Stroke Cerebrovasc Dis*. 2014;23:114-122 | Does not investigate multiple thresholds |
| Hana T, Iwama J, Yokosako S, Yoshimura C, Arai N, Kuroi Y, et al. Sensitivity of ct perfusion for the diagnosis of cerebral infarction. *J Med Invest*. 2014;61:41-45 | Does not investigate multiple thresholds |
| Thierfelder KM, von Baumgarten L, Löchelt AC, Meinel FG, Armbruster M, Beyer SE, et al. Diagnostic accuracy of whole-brain computed tomographic perfusion imaging in small-volume infarctions. *Invest Radiol*. 2014;49:236-242 | Does not investigate multiple thresholds |
| Lin, K., Rapalino, O., Lee, B., Do, K. G., Sussmann, A. R., Law, M., & Pramanik, B. K. (2009). Correlation of volumetric mismatch and mismatch of Alberta Stroke Program Early CT Scores on CT perfusion maps. *Neuroradiology*, *51*(1), 17-23. | Does not investigate multiple thresholds |
| Koenig M, Kraus M, Theek C, Klotz E, Gehlen W, Heuser L. Quantitative assessment of the ischemic brain by means of perfusion-related parameters derived from perfusion ct. *Stroke*. 2001;32:431-437 | Does not co-register images |
| Kameda K, Uno J, Otsuji R, Ren N, Nagaoka S, Maeda K, et al. Optimal thresholds for ischemic penumbra predicted by computed tomography perfusion in patients with acute ischemic stroke treated with mechanical thrombectomy. *Journal of NeuroInterventional Surgery*. 2018;10:279-284 | Does not co-register images |
| Sparacia G, Iaia A, Assadi B, Lagalla R. Perfusion ct in acute stroke: Predictive value of perfusion parameters in assessing tissue viability versus infarction. *Radiol Med*. 2007;112:113-122 | Does not co-register images |
| Copen WA, Morais LT, Wu O, Schwamm LH, Schaefer PW, González RG, et al. In acute stroke, can ct perfusion-derived cerebral blood volume maps substitute for diffusion-weighted imaging in identifying the ischemic core? *PLoS One*. 2015;10:e0133566 | Does not co-register images |
| Qiao Y, Zhu G, Patrie J, Xin W, Michel P, Eskandari A, et al. Optimal perfusion computed tomographic thresholds for ischemic core and penumbra are not time dependent in the clinically relevant time window. *Stroke*. 2014;45:1355-1362 | Does not co-register images |
| Mokin M, Levy EI, Saver JL, Siddiqui AH, Goyal M, Bonafé A, et al. Predictive value of rapid assessed perfusion thresholds on final infarct volume in swift prime (solitaire with the intention for thrombectomy as primary endovascular treatment). *Stroke*. 2017;48:932-938 | Does not co-register images |
| Pan J, Zhang J, Huang W, Cheng X, Ling Y, Dong Q, et al. Value of perfusion computed tomography in acute ischemic stroke: Diagnosis of infarct core and penumbra. *J Comput Assist Tomogr*. 2013;37:645-649 | Does not co-register images |
| Albers GW, Goyal M, Jahan R, Bonafe A, Diener HC, Levy EI, et al. Ischemic core and hypoperfusion volumes predict infarct size in swift prime. *Ann Neurol*. 2016;79:76-89 | Included patients who had MRP as index test |
| d'Esterre CD, Roversi G, Padroni M, Bernardoni A, Tamborino C, De Vito A, et al. Ct perfusion cerebral blood volume does not always predict infarct core in acute ischemic stroke. *Neurol Sci*. 2015;36:1777-1783 | Does not investigate multiple thresholds |
| Rao V, Christensen S, Yennu A, Mlynash M, Zaharchuk G, Heit J, et al. Ischemic core and hypoperfusion volumes correlate with infarct size 24 hours after randomization in defuse 3. *Stroke*. 2019;50:626-631 | Included patients who had MRP as index test |
| Touho H, Karasawa J. Evaluation of time-dependent thresholds of cerebral blood flow and transit time during the acute stage of cerebral embolism: A retrospective study. *Surg Neurol*. 1996;46:135-145; discussion 145-136 | Does not co-register images |
| Suzuki Y, Nakajima M, Ikeda H, Abe T. Evaluation of hyperacute stroke using perfusion computed tomography. *Neurol Med Chir (Tokyo)*. 2005;45:333-343; discussion 341-333 | Does not investigate multiple thresholds |
| Hagiwara H, Nakamura H, Igarashi H, Katayama Y. Predicting the fate of acute ischemic lesions using perfusion computed tomography. *J Comput Assist Tomogr*. 2008;32:645-650 | Does not co-register images |
| Fainardi E, Busto G, Rosi A, Scola E, Casetta I, Bernardoni A, et al. Tmax volumes predict final infarct size and functional outcome in ischemic stroke patients receiving endovascular treatment. Ann Neurol. 2022 | Does not co-register images |
| Giammello F, De Martino SRM, Simonetti L, Agati R, Battaglia S, Cirillo L, et al. Predictive value of tmax perfusion maps on final core in acute ischemic stroke: An observational single-center study. *Radiol Med*. 2022;127:414-425 | Does not investigate multiple thresholds |
| Muehlen I, Sprugel M, Hoelter P, Hock S, Knott M, Huttner HB, et al. Comparison of two automated computed tomography perfusion applications to predict the final infarct volume after thrombolysis in cerebral infarction 3 recanalization. *Stroke*. 2022;53:1657-1664 | Does not co-register images |
| Huynh DC, Parsons MW, Wintermark MM, Vagal A, d'Esterre CD, Vitorino RR, et al. Can ct perfusion accurately assess infarct core? *Neurovascular Imaging*. 2016;2 | Optimal threshold not documented |
| Chen C, Parsons MW, Levi CR, Spratt NJ, Lin L, Kleinig T, et al. What is the "optimal" target mismatch criteria for acute ischemic stroke? *Front Neurol*. 2020;11:590766 | Does not co-register images |
| Koopman MS, Berkhemer OA, Geuskens R, Emmer BJ, van Walderveen MAA, Jenniskens SFM, et al. Comparison of three commonly used ct perfusion software packages in patients with acute ischemic stroke. *J Neurointerv Surg*. 2019;11:1249-1256 | Does not co-register images |
| Xiong Y, Huang CC, Fisher M, Hackney DB, Bhadelia RA, Selim MH. Comparison of Automated CT Perfusion Softwares in Evaluation of Acute Ischemic Stroke. J Stroke Cerebrovasc Dis. 2019 Dec;28(12):104392. doi: 10.1016/j.jstrokecerebrovasdis.2019.104392. Epub 2019 Sep 25. PMID: 31562038. | Does not co-register images |
| Schaefer PW, Souza L, Kamalian S, Hirsch JA, Yoo AJ, Kamalian S, Gonzalez RG, Lev MH. Limited reliability of computed tomographic perfusion acute infarct volume measurements compared with diffusion-weighted imaging in anterior circulation stroke. Stroke. 2015 Feb;46(2):419-24. doi: 10.1161/STROKEAHA.114.007117. Epub 2014 Dec 30. PMID: 25550366; PMCID: PMC4308477. | Does not co-register images |
| Sakai Y, Delman BN, Fifi JT, Tuhrim S, Wheelwright D, Doshi AH, et al. Estimation of ischemic core volume using computed tomographic perfusion. Stroke. 2018;49(10): 2345–52 | Does not investigate multiple thresholds |
| Schaefer PW, Barak ER, Kamalian S, Gharai LR, Schwamm L, Gonzalez RG, et al. Quantitative assessment of core/penumbra mismatch in acute stroke: CT and MR perfusion imaging are strongly correlated when sufficient brain volume is imaged. Stroke. 2008; 39(11):2986–92. | Does not co-register images |
| Rudilosso S, Laredo C, Vivancos C, Urra X, Llull L, Renú A, Obach V, Zhao Y, Moreno JL, Lopez-Rueda A, Amaro S, Chamorro Á. Leukoaraiosis May Confound the Interpretation of CT Perfusion in Patients Treated with Mechanical Thrombectomy for Acute Ischemic Stroke. AJNR Am J Neuroradiol. 2019 Aug;40(8):1323-1329. doi: 10.3174/ajnr.A6139. Epub 2019 Jul 25. PMID: 31345941; PMCID: PMC7048478. | Does not co-register images |
| Rudilosso S, Urra X, San Román L, Laredo C, López-Rueda A, Amaro S, et al. Perfusion deficits and mismatch in patients with acute lacunar infarcts studied with whole-brain CT perfusion. AJNR Am J Neuroradiol. 2015; 36(8):1407–12. | Does not investigate multiple thresholds |
| Hoving JW, Marquering HA, Majoie C, Yassi N, Sharma G, Liebeskind DS, et al. Volumetric and spatial accuracy of computed tomography perfusion estimated ischemic core volume in patients with acute ischemic stroke. *Stroke*. 2018;49:2368-2375 | Does not investigate multiple thresholds |
| Rava RA, Snyder KV, Mokin M, Waqas M, Allman AB, Senko JL, et al. Assessment of a Bayesian Vitrea CT perfusion analysis to predict final infarct and penumbra volumes in patients with acute ischemic stroke: a comparison with RAPID. AJNR Am J Neuroradiol. 2020;41(2):206–12. | Does not investigate multiple thresholds |
| Karamchandani RR, Rhoten JB, Strong D, Chang B, Defilipp G, Bernard J, et al. Computed tomography perfusion core infarct measurement compared to diffusion-weighted magnetic resonance imaging in patients with revascularization of anterior circulation, large artery occlusion ischemic stroke. SN Compr Clin Med. 2020;2(12):2730–7 | Does not co-register images |
| Ostman C, Garcia-Esperon C, Lillicrap T, Tomari S, Holliday E, Levi C, et al. Multimodal computed tomography increases the detection of posterior fossa strokes compared to brain non-contrast computed tomography. Front Neurol. 2020;11 | Does not investigate multiple thresholds |
| Lin L, Bivard A, Kleinig T, Spratt NJ, Levi CR, Yang Q, et al. Correction for delay and dispersion results in more accurate cerebral blood flow ischemic core measurement in acute stroke. Stroke. 2018;49(4):924–30 | Does not investigate multiple thresholds |
| Kasasbeh AS, Christensen S, Parsons MW, Campbell B, Albers GW, Lansberg MG. Artificial neural network computer tomography perfusion prediction of ischemic core. Stroke. 2019;50(6):1578–81. | Integrated data from all parameters |
